# Polyimide aerogels for ballistic impact protection

**DOI:** 10.1038/s41598-022-18247-z

**Published:** 2022-08-17

**Authors:** Sadeq Malakooti, Stephanie L. Vivod, Michael Pereira, Charles R. Ruggeri, Duane M. Revilock, Runyu Zhang, Haiquan Guo, Daniel A. Scheiman, Linda S. McCorkle, Hongbing Lu

**Affiliations:** 1 Materials and Structures Division, NASA Glenn Research Center, 21000 Brookpark Road, Cleveland, OH 44135 USA; 2 Department of Mechanical Engineering, The University of Texas at Dallas, Richardson, TX 75080 USA; 3 Universities Space Research Association, 7178 Columbia Gateway Drive, Columbia, MD 21046 USA

**Keywords:** Mechanical properties, Mechanical engineering

## Abstract

The ballistic performance of edge-clamped monolithic polyimide aerogel blocks (12 mm thickness) has been studied through a series of impact tests using a helium-filled gas gun connected to a vacuum chamber and a spherical steel projectile (approximately 3 mm diameter) with an impact velocity range of 150–1300 m s^−1^. The aerogels had an average bulk density of 0.17 g cm^−3^ with high porosity of approximately 88%. The ballistic limit velocity of the aerogels was estimated to be in the range of 175–179 m s^−1^. Moreover, the aerogels showed a robust ballistic energy absorption performance (e.g., at the impact velocity of 1283 m s^−1^ at least 18% of the impact energy was absorbed). At low impact velocities, the aerogels failed by ductile hole enlargement followed by a tensile failure. By contrast, at high impact velocities, the aerogels failed through an adiabatic shearing process. Given the substantially robust ballistic performance, the polyimide aerogels have a potential to combat multiple constraints such as cost, weight, and volume restrictions in aeronautical and aerospace applications with high blast resistance and ballistic performance requirements such as in stuffed Whipple shields for orbital debris containment application.

## Introduction

Orbital debris are residues from launched objects that are still in orbit around the earth^1^. The most common source of the debris comes from space object explosions resulting in particles typically in millimeter sizes^2^. Due to their high velocities, for decades, orbital debris has been considered one of the most important threats to space flight safety^3^. The impact velocity is in the range of 7–10 km s^−1^ at low earth orbit^4^. This threat is now even bigger with increasing global space activity^5^. Therefore, developing a lightweight but effective shield system against hypervelocity particles is crucial for any space exploration mission.

In this regard, Fred Whipple in the 1940s proposed an orbital debris shield system for spacecrafts consisting of a sacrificial thin bumper sheet and a thick rear wall separated by a certain distance^6,7^. The role of the sacrificial bumper sheet is to break up the debris and make a debris cloud. The thickness of the rear wall should be enough to withstand the debris cloud blast momentum. In addition, in order to enhance the shielding performance of the Whipple shields, they are typically stuffed with high-strength fabrics such as several layers of Nextel and Kevlar fabrics^7^.

Currently, stuffed Whipple shields are primarily used on the International Space Station (ISS) for orbital debris containment^8^. Typically on the ISS, the bumpers are 2-mm thick Al 6061-T6, the rear walls are 4.8-mm thick Al 2219-T87 or Al 2219-T851 and the stuffing materials are 6 layers of Kevlar 29 style 710 with 6 layers of Nextel AF62 fabrics at different distances from each other^7,9^. The total distance between the bumper wall and the rear wall is more than 11 cm with no material spacing in between. The current design configuration is essentially based on maximizing the strength-to-weight ratio of the shield interior. However, this design is still bulky and can be improved with further reductions not only in the total weight but also in the total shield volume. One prominent approach is to use low-density impact-resistant materials in the stand-off spacing of the stuffed Whipple shields to decelerate/capture secondary debris clouds in their microstructures^10^. Using low-density impact-resistant materials as an interior shield augmentation offers an increase in the ballistic performance of the stuffing materials. This also allows for a reduction in the thickness of the heavy metal rear wall of the shield system as well as a reduction in the stand-off spacing between the Whipple bumper and backplate which would potentially lead to significant mass and volume savings.

Historically, hypervelocity cosmic particles such as comet and interstellar dust particles at a typical velocity of 6 km s^−1^ were captured by silica aerogels and returned to earth through the Stardust NASA mission^11,12^. Some years later, silica aerogels were also used at low earth orbit at the International Space Station as a passive detector to detect and quantify craters^13^. Upon returning the aerogel samples to earth, they contained a lot of debris. It is worth mentioning that although the size of the collected particles and debris were only a few tens of microns, these studies are indicative of high potential ballistic containment feasibility in aerogel materials. As silica aerogels are extremely fragile and their mechanical properties including Young’s and flexural moduli are so low, their hypervelocity microparticle containment capability is attributed to their highly tortuous microstructures formed by a random agglomeration of silica nanoparticles as well as their high specific surface areas. However, a one millimeter-sized debris particle has 1 billion times more kinetic energy than one micron-sized spherical particle at a similar density and impact velocity. Therefore, fragile silica aerogels are not effective as a debris containment remediation application for millimeter-sized debris due to their weak mechanical properties^14^.

Alternatively, polymer aerogels can be synthesized with a highly tortuous and mesoporous microstructure with excellent mechanical properties at a bulk density similar to the ones corresponding to silica aerogels^15–20^. Polymer aerogels exhibit high ductility with mechanical properties such as Young’s modulus orders of magnitude higher than the corresponding values for silica aerogels^21–24^. Polymer aerogel emergence led to a paradigm shift in our view on the mechanics of aerogel materials^25^. Among many polymer aerogels, high-performance polyimide aerogels have been implemented in various aeronautics and aerospace applications due to their strong mechanical properties and their excellent structural integrity at high temperatures^15,26–29^.

**Figure 1:**
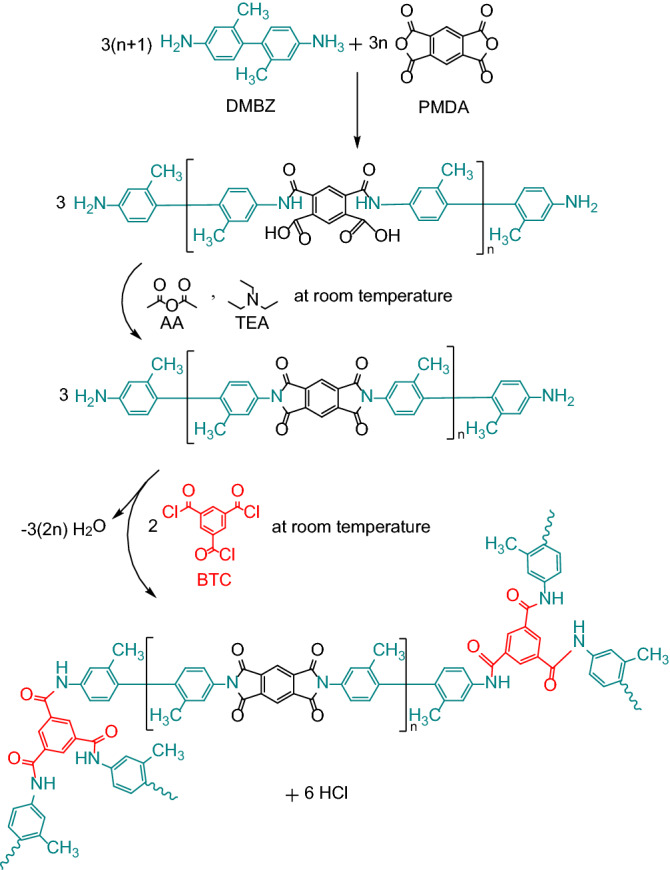
Chemical synthesis of the polyimide aerogels.

With this background in mind, the primary intent of this work is to investigate the ballistic performance of a class of mechanically strong polyimide aerogels through a series of ballistic impact tests at various velocities. The experiments were conducted using a helium-filled gas gun connected to a vacuum chamber and a spherical steel projectile (3.175 mm diameter). The results of this work will help in the design and manufacturing of new advanced stuffed Whipple shield systems for future space exploration missions. Accordingly, the polyimide gels were synthesized through a sol–gel process using stoichiometric amounts of pyromellitic dianhydride (PMDA) and 2,2′-dimethylbenzidine (DMBZ) in N-methyl-2-pyrrolidone (NMP) to create a solution of polyamic acid oligomers. These oligomers were imidized by chemical means at room temperature using acetic anhydride (AA) as a water scavenger and triethyl amine (TEA) as a base catalyst. The imidized oligomers were further cross-linked using commercially available 1,3,5-benzenetricarbonyl trichloride (BTC). The reaction pathway of the polyimide aerogel synthesis is shown in Fig. 1. In the final step, the gels were solvent-exchanged into high-purity *tert*-butanol, which was then amorphously and isochorically frozen below room temperature and subsequently sublimed under a flow of dry gas at atmospheric pressure, eliminating the need for a vacuum vessel.

## Results and discussion

### Material characterization

Twelve polyimide aerogel samples with identical chemical formulations (10 w% polymer concentration with $n=40$, the number of repeat units) with nominal dimensions of 76.2 mm × 76.2 mm × 12 mm were prepared for this study. The bulk density, porosity, shrinkage, and the actual thickness of the polyimide aerogel samples are listed in Table 1. The average bulk density of the samples was 0.17 g cm^−3^. The density variation (12% standard deviation) is due to the change in the sample’s sublimation rates at different dryer locations. Moreover, no sign of shrinkage was observed during the aging and solvent exchange processes. The average total linear shrinkage of the samples relative to their molds was 18.8%. The skeletal density of the aerogels was measured to be 1.46 g cm^−3^. Using the skeletal and bulk densities, the porosity of the samples was calculated to be 88.3%. At the lowest shrinkage (the PI-3 with 15% shrinkage, refer to Table 1), the sample has a porosity close to 90%. At the highest shrinkage (the PI-12 with 24% shrinkage, refer to Table 1), the sample’s porosity is 85.6%.

**Table 1:** General material properties and thickness of the polyimide aerogel blocks.

Name	Bulk density, *ρ*_b_ (g cm^−3^)	Porosity, Π (% v/v)^a^	Linear shrinkage (%)^b^	Thickness (cm)
PI-1	0.19	86.99	22	1.15
PI-2	0.16	89.05	18	1.17
PI-3	0.15	89.73	15	1.50
PI-4	0.16	89.05	17	1.25
PI-5	0.16	89.05	17	1.20
PI-6	0.16	89.05	18	1.20
PI-7	0.16	89.05	17	1.10
PI-8	0.19	86.99	22	1.20
PI-9	0.15	89.73	16	1.20
PI-10	0.16	89.05	17	1.18
PI-11	0.2	86.31	23	1.11
PI-12	0.21	85.62	24	1.09
Average	**0.17 ± 0.02**	**88.30 ± 1.41**	**18.83 ± 3.04**	**1.20 ± 0.11**

Significant values are in [bold].

^a^Porosity = 100 × [(*ρ*_s_−*ρ*_b_)/*ρ*_s_], where *ρ*_s_ is 1.46 ± 0.08 g cm^−3^, the skeletal density measured by Helium pycnometry.

^b^Shrinkage = 100 × (mold diagonal− sample diagonal)/(mold diagonal).

**Figure 2:**
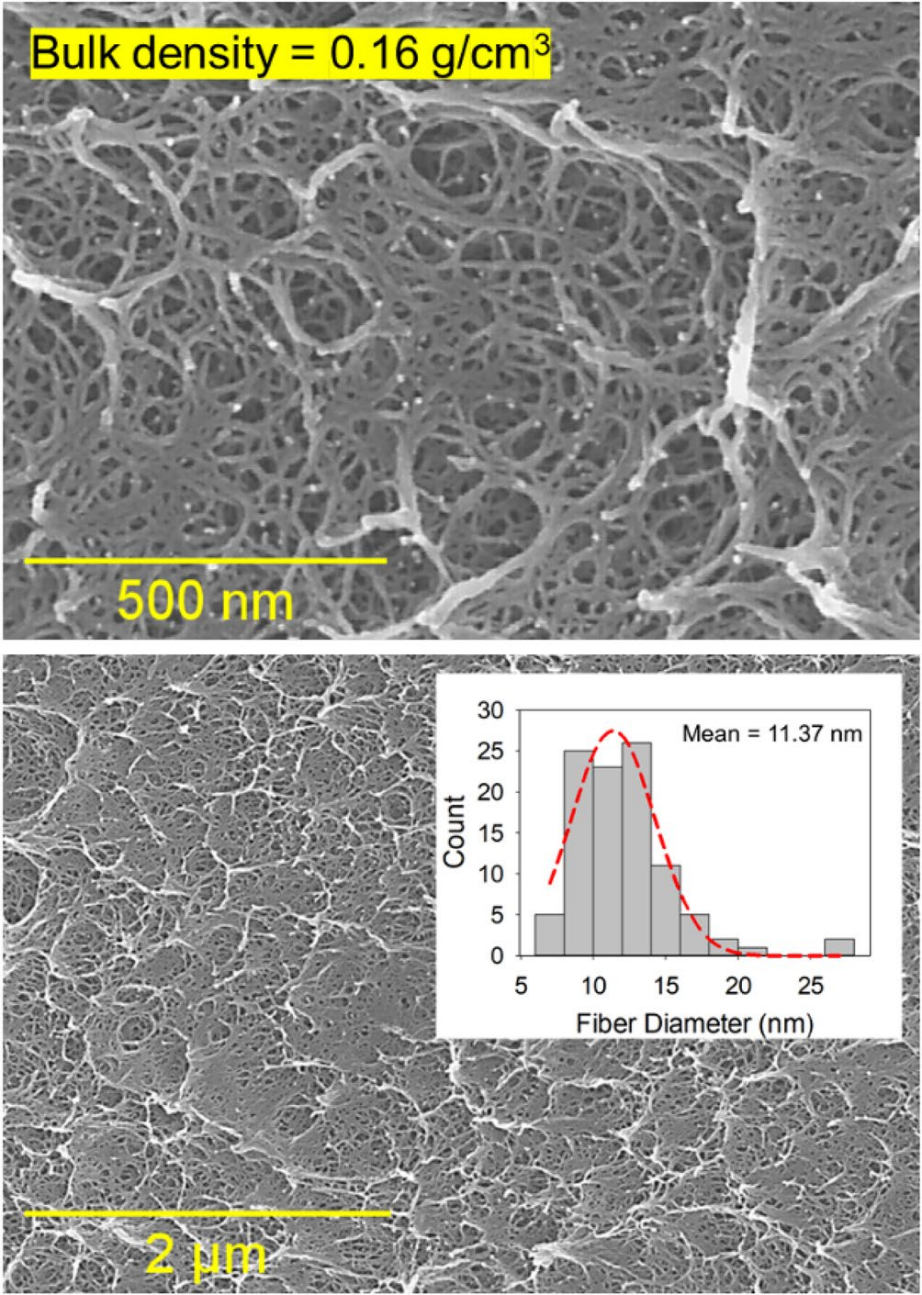
Representative SEM micrographs at different magnifications for the polyimide aerogels with 0.16 g/cm^3^ bulk density ($n=$ 40, 10% w/w). The fiber diameter size distribution of the aerogel is also shown in the inset.

The morphology of the aerogels was studied using a scanning electron microscope (SEM). Figure 2 shows the SEM images of a polyimide aerogel sample (PI-7) with a bulk density of 0.16 g/cm^3^ at different magnifications. The aerogel micrographs exhibit a highly entangled and tortuous fibrous microstructure with an average fiber diameter of 11.37 nm. The fiber diameter size distribution is shown as an inset in Fig. 2. Microstructure plays a significant role in the energy dissipation performance. For glassy polymers, the main energy dissipation mechanism comes from intermolecular disentanglement and molecular scission^30^. Therefore, polymeric systems with highly entangled fibrous microstructures, as in the case of this study, typically outperform their counterparts with particulate microstructures for mechanical energy absorption applications including impact energy mitigation.

**Figure 3:**
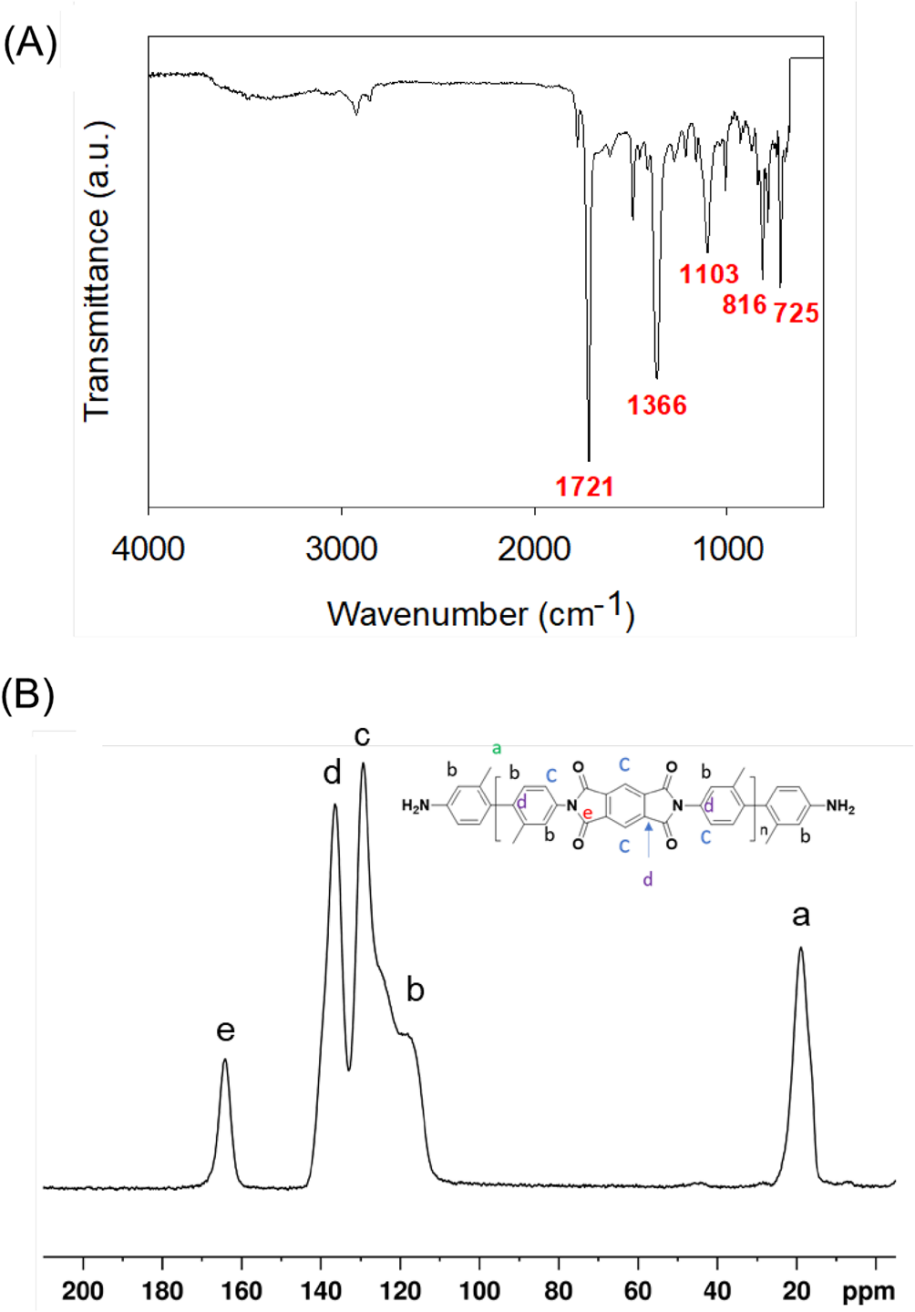
(**A**) Representative FTIR and (**B**) Solid-state CPMAS ^13^C NMR spectra of polyimide aerogels.

Using FTIR spectroscopy, the chemical bonds of the polyimide aerogels were identified. Figure 3A shows the FTIR spectrum of a polyimide aerogel sample (PI-6). The identified absorptions (cm^−1^) are as follows: the imide carbonyl (C=O) at 1721 (s), the imide C-N at 1366 (s), the benzenes substitutes from PMDA and DMBZ at 1103 (m) and 816 (m), and the imide at 725 (m)^31^. Figure 3B displays the representative ^13^C NMR spectrum of the polyimide aerogels. The spectrum has a peak at 167.1 ppm, which is indicative of the imide carbonyl, the aromatic peaks between 110 and 140 ppm, and the peak at 18.7 ppm are assigned to the methyl groups from DMBZ^32^.

**Figure 4:**
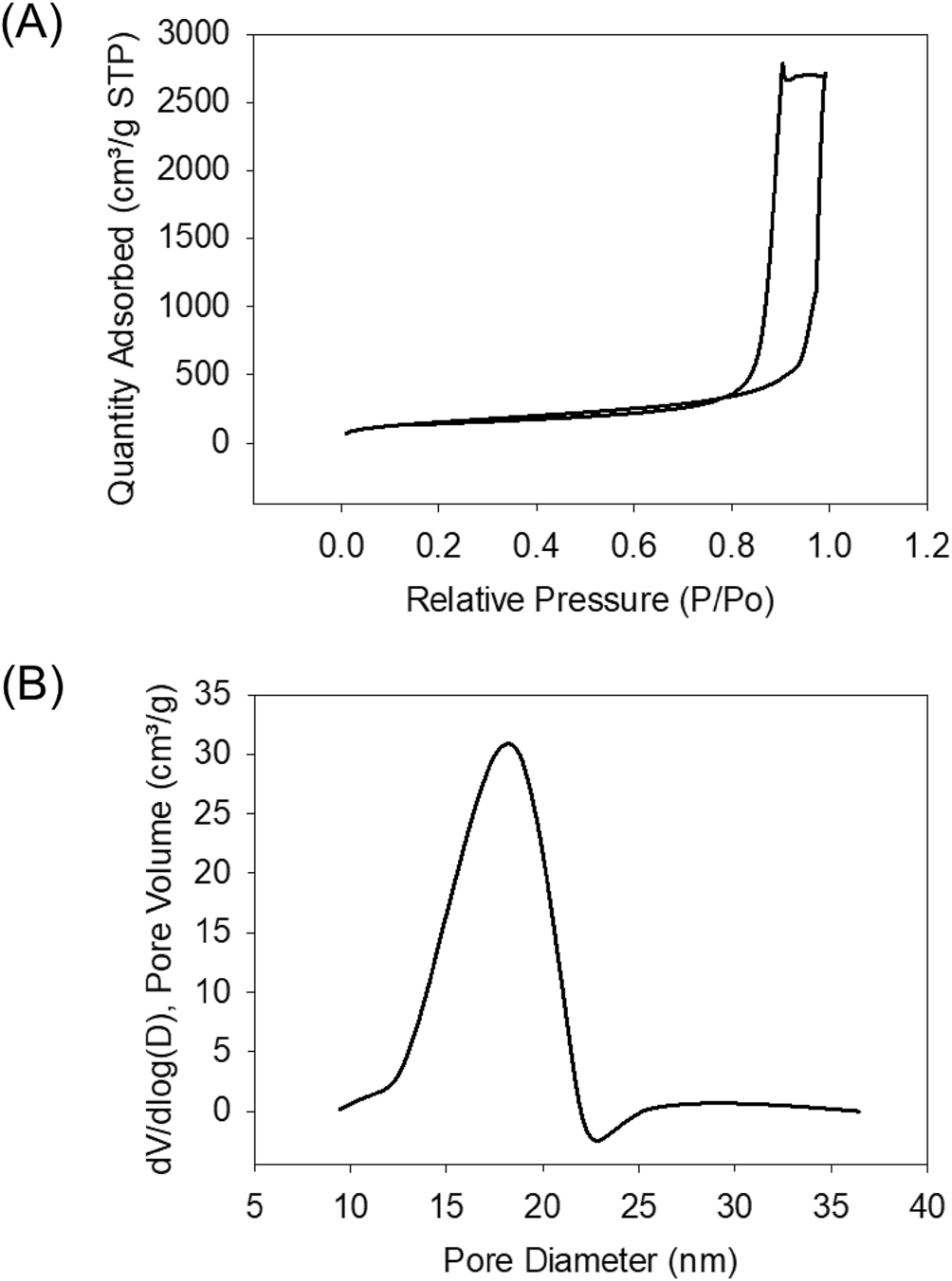
(**A**) Representative N_2_-sorption isotherm plot at 77 K, and (**B**) BJH pore size distribution of polyimide aerogels.

The porous structure of the polyimide aerogels was studied through N_2_-sorption measurements as shown in Fig. 4A and B^33^. The aerogels are mainly mesoporous with a high specific surface area of about 566 m^2^ g^−1^. The average pore size of the aerogels using BJH desorption is 16.84 nm (check Fig. 4B). The large surface area is indicative of a narrow pore size distribution in agreement with Fig. 4B.

**Figure 5:**
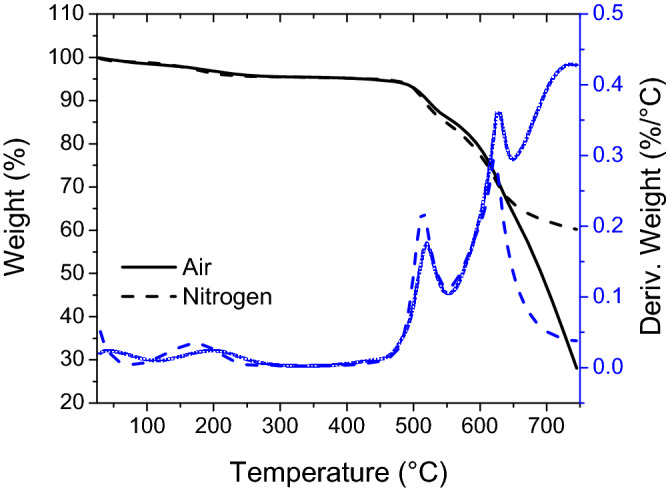
Representative TGA and DTG plots of the polyimide aerogels.

The thermal degradation properties of the aerogels were also studied utilizing thermogravimetric analysis (TGA) and derivative thermogravimetry (DTG) as shown in Fig. 5. A slight mass loss (approximately 5%) is existed at the beginning of TGA signal at ~ 300 °C. This might be due to the removal of moisture or any trapped solvent from the ambient pressure drying process. The onset of decomposition appeared between 459 and 507 °C. The rate of mass loss significantly increases after the decomposition temperature (also a significant change in DTG values). The solid weight residues at 700 °C are approximately 50% and 65% under air and nitrogen atmosphere, respectively.

**Figure 6:**
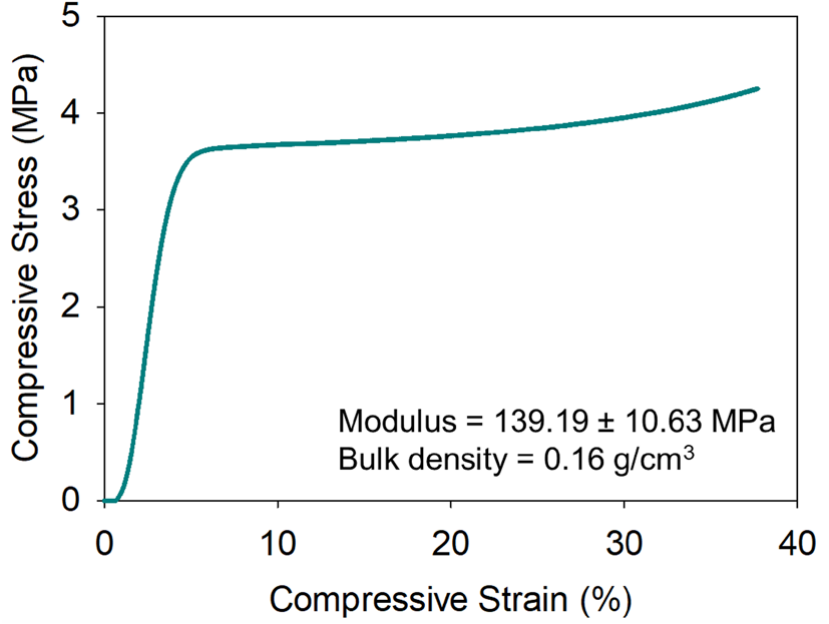
Typical quasi-static uniaxial compressive stress–strain response of the polyimide aerogel at 0.16 g cm^−3^ bulk density.

The Young’s modulus of the aerogels at a low strain rate (i.e., 0.01 s^–1^) was also measured at room temperature. The typical stress–strain curve of the polyimide aerogel samples in compression is shown in Fig. 6. The compressive behavior of the polyimide aerogels follows three stages: elastic deformation, compaction, and densification. No buckling was observed in the sample under compression. The Young’s modulus of the aerogels at 0.16 g cm^−3^ was found to be about 139 MPa. This is considered a high modulus for such a low bulk density material^25^. The specific modulus of these aerogels (i.e., modulus per unit density) is 0.87 × 10^6^ m^2^ s^−2^ which is three orders of magnitude higher than the corresponding values for typical high density polymeric foams such as latex foam (0.0002 × 10^6^ m^2^ s^−2^) and twice as high as state-of-the-art aluminum foams, such as Duocel® (0.47 × 10^6^ m^2^ s^−2^), at a similar bulk density.

### Ballistic performance

**Figure 7:**
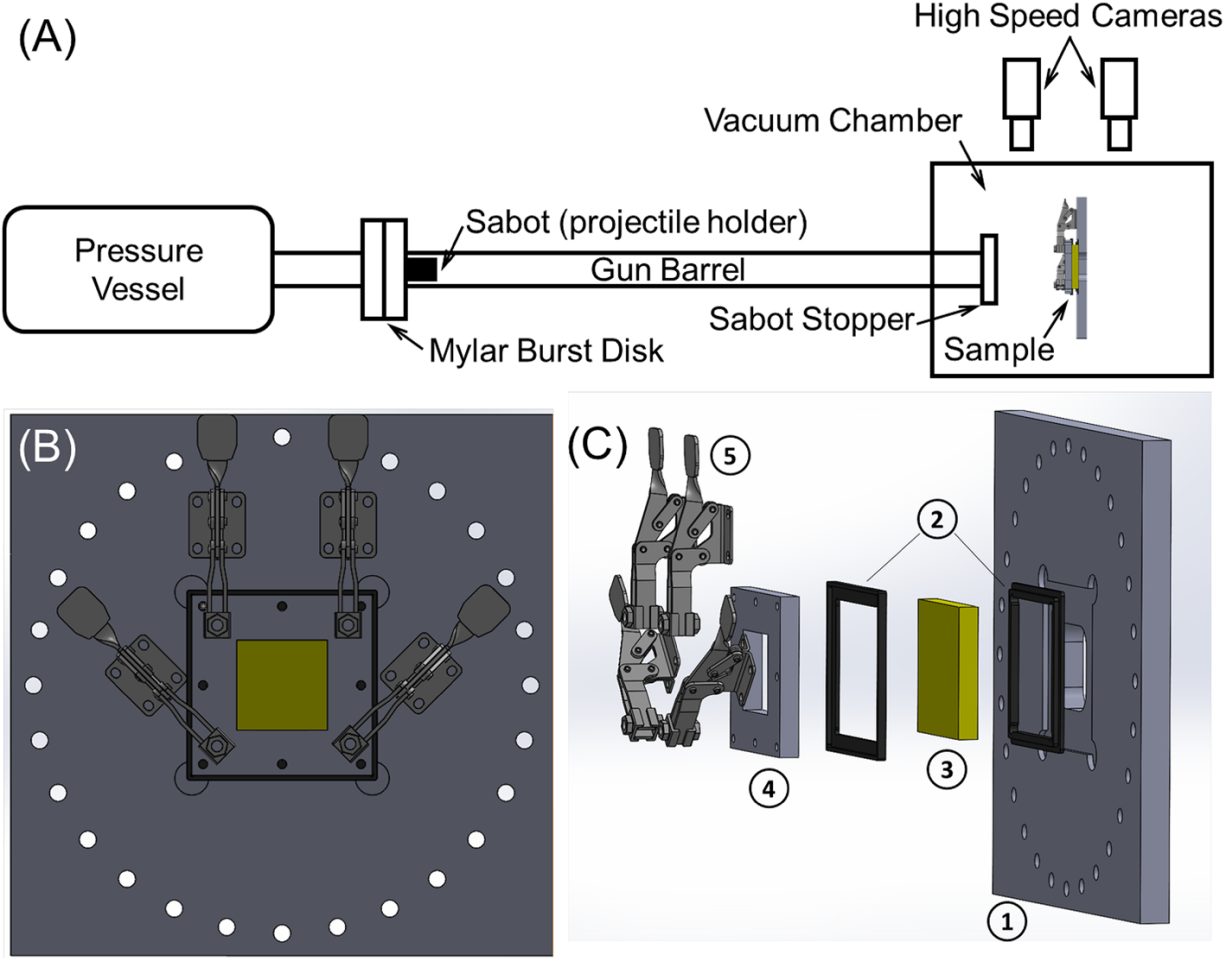
(**A**) Sketch showing the principal components of the helium-filled gas gun connected to a vacuum chamber utilized in this study; (**B**) front view of the specimen clamping fixture with an open window of 2 in by 2 in; and (**C**) exploded view of the specimen clamping fixture comprising of different components: (1) base plate, (2) rubber seals, (3) test specimen, (4) face support, and (5) mechanical clamps.

The ballistic impact response of the aerogel blocks was studied by means of a helium-filled gas gun connected to a vacuum chamber^34^. Figure 7A shows the schematic of the experimental setup. The gun propelled a sabot carrying 3.175 mm diameter spherical steel projectiles. The aerogel samples were edge-clamped using a clamping fixture. Figure 7B and C show the components of the clamping fixture. The sample test window size is 50.8 mm by 50.8 mm.

The measured projectile impact and exit velocities and the aerogels absorbed energy percentage at different velocities are listed in Table 2. The projectile impact velocities are in the range of 150–1300 m s^−1^. The percent absorbed energy by the aerogel blocks was calculated using the ratio of $\left( {v_{i}^{2} - v_{e}^{2} } \right)/v_{i}^{2}$, where $v_{i}$ and $v_{e}$ are the projectile impact and exit velocities, respectively.

**Table 2:** Ballistic impact test results for the polyimide aerogel blocks.

Name	Areal density, *ρ*_a_ (g cm^−2^)	Impact velocity, *v*_i_ (m ^−1^)	Exit velocity, *v*_e_ (m s^−1^)	Absorbed energy, (%)
PI-1	0.22	1283.05	1160.18	18.24
PI-2	0.19	1091.16	1018.95	12.80
PI-3	0.23	1237.40	1145.32	14.33
PI-4	0.20	466.24	369.21	37.29
PI-5	0.19	435.22	371.63	27.09
PI-6-A	**0.19**	**160.92**	**0**	**100**
PI-6-B	**0.19**	**174.09**	**0**	**100**
PI-7	0.17	179.21	31.61	96.89
PI-8	**0.23**	**171.53**	**0**	**100**
PI-9	0.18	921.65	874.03	10.07
PI-10	0.19	660.51	577.28	23.61
PI-11	0.22	875.57	819.38	12.42
PI-12	0.23	675.88	584.39	25.24

Significant values are in [bold].

**Figure 8:**
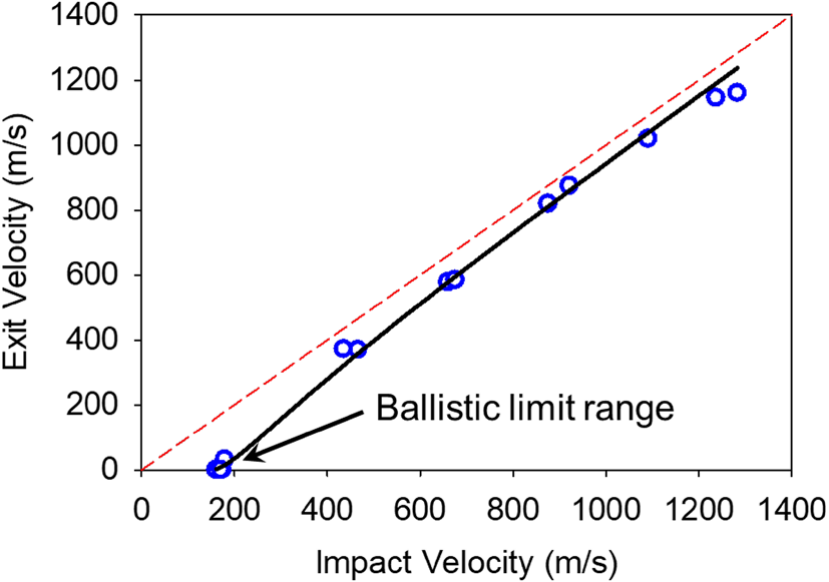
Measured projectile exit velocity ${v}_{e}$ as a function projectile impact velocity ${v}_{i}$.

Figure 8 shows the projectile exit velocity as a function of the projectile impact velocity. Below the ballistic limit velocity, the aerogel blocks absorb all the kinetic energy of the projectile. Using Table 1 and Fig. 8, the ballistic limit velocity of the aerogels is estimated to be between 175 and 179 m s^−1^.

**Figure 9:**
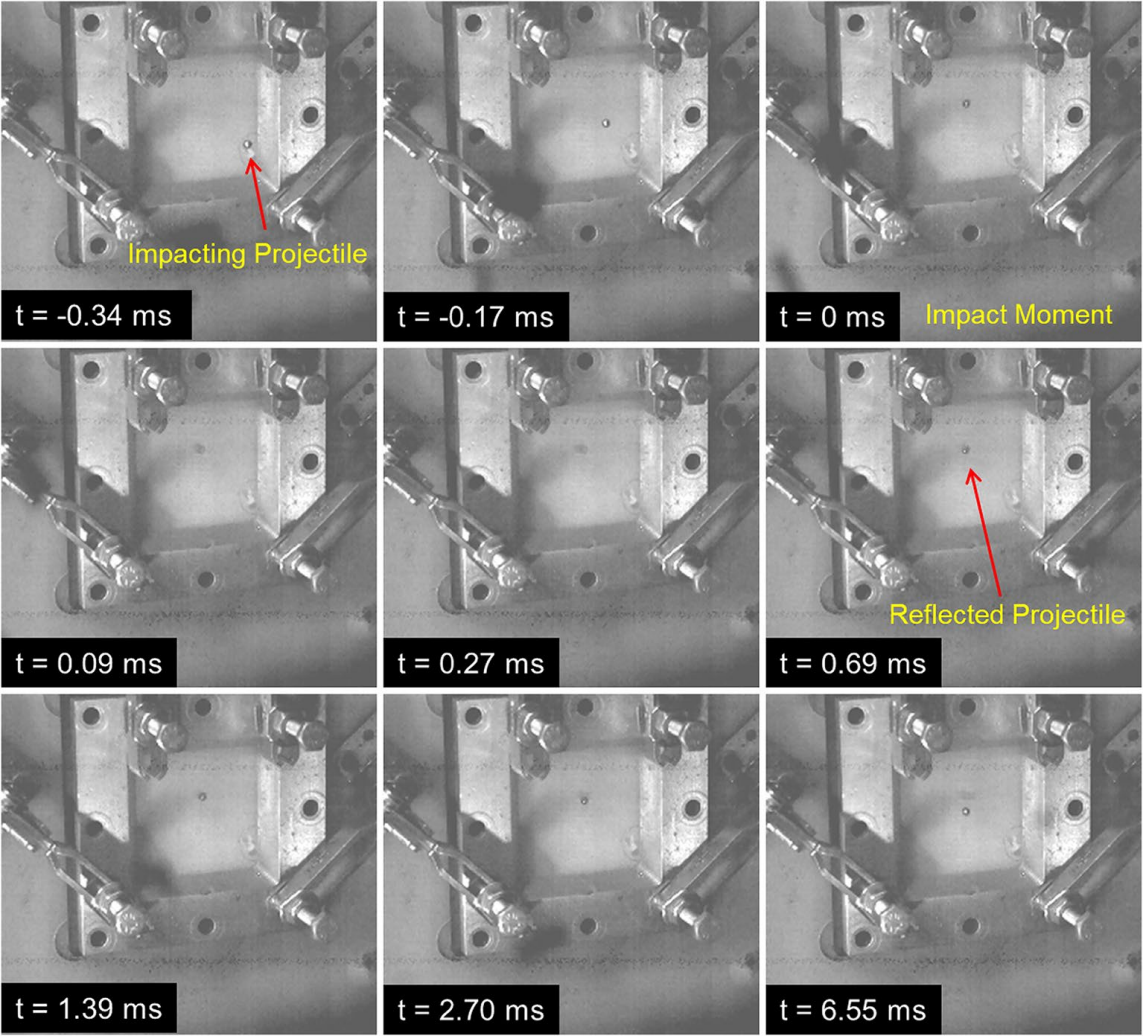
High-speed photographic sequence of the impact of the polyimide aerogel block (PI-6) by a projectile at the impact velocity of 174 m s^−1^. Time relative to the impact moment when the projectile touches the front face of the aerogel block is also indicated for each frame.

An example of a sequence of high-speed photographs associated with the impact of the spherical projectile at 174 m s^−1^ impact velocity against a polyimide aerogel block (PI-6) is shown in Fig. 9. The time for each photograph is marked in the figures with $t=$ 0 taken as the instant of the impact against the front face of the aerogel block. For this sample, at this impact velocity, the projectile penetrates the front face of the aerogel block and then reflects off from the block at $t=$ 0.69 ms. It is noted that the projectile at 171.53 m s^−1^ impact velocity was stopped by and trapped in the PI-8 aerogel block. A footage associated with the impact event on the PI-6 sample is provided in the Supporting Information (Movie S1).

**Figure 10:**
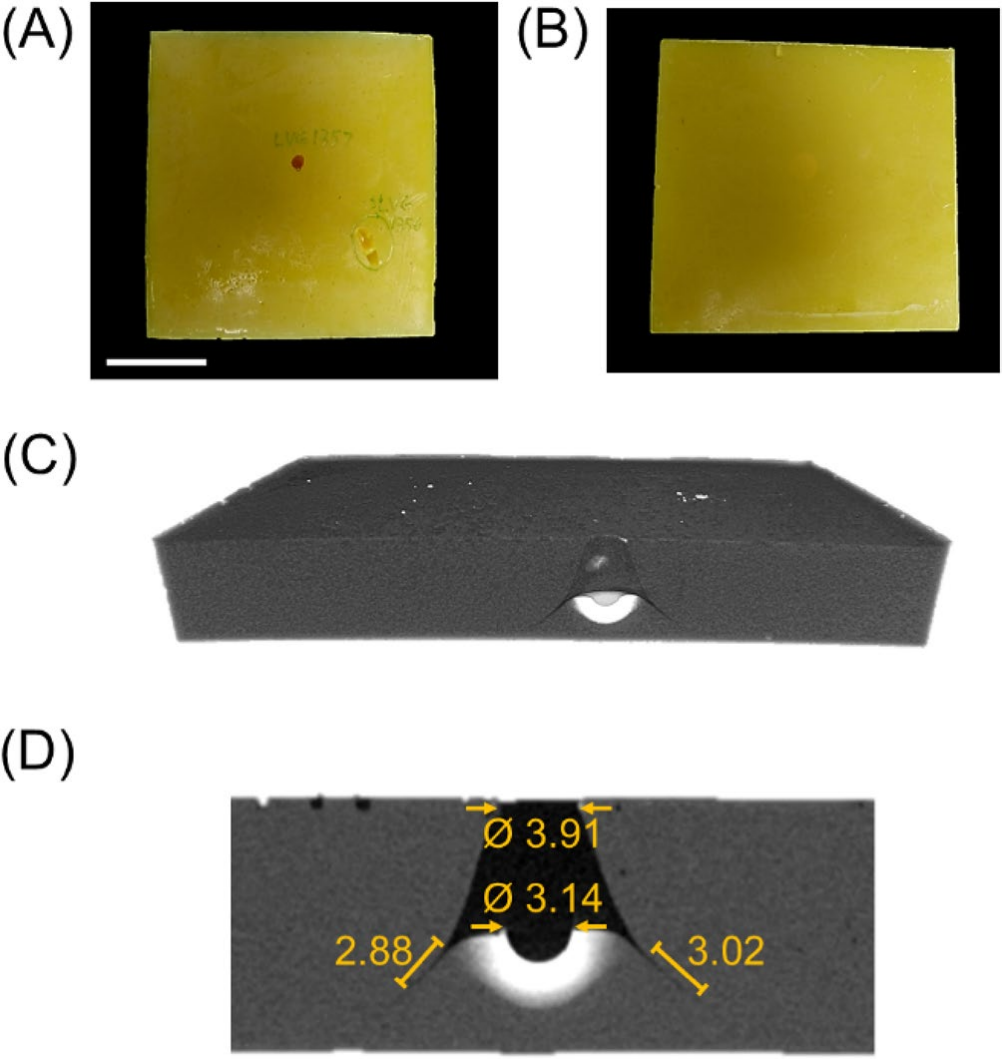
(**A**) The front and (**B**) the rear faces of the PI-6 aerogel block after impact; (**C**) A cross-section 3D rendering of the impacted PI-6 aerogel block; and (**D**) A zoomed-in view from the cross-section of the PI-6 impact zone (dimensions are in mm). The scale bar is 1 in.

The optical images taken from the front and rear faces of the sample associated with Fig. 9 (PI-6) are also shown in Fig. 10A and B, respectively. During the impact process, an exchange of energy takes place between the projectile and the target. The kinetic energy of the projectile is exchanged partly with an increase in the internal energy and material deformation (resulting in material failure) in the target or become lost in the form of eroded material. As the projectile is sufficiently stronger and stiffer than the aerogel, the projectile kinetic energy reduction is only attributed to the aerogel’s deformation and failure. The aerogel’s material failure was studied through a series of X-ray micro-computed tomography (µ-CT) of the impacted aerogel blocks. Movie S2 in the Supporting Information shows cross-sectional slices of the CT scanned volumetric image of the PI-6 sample. Figure 10C shows a 3D rendering of the impacted PI-6 aerogel block. This image shows a localized material damage for the impact zone of the PI-6 aerogel block. An enlarged view from the cross-section of the PI-6 impact zone is also shown in Fig. 10D. High-density zones (compressed material) are shown brighter than intact material zones. The aerogels at low impact velocities experienced a ductile failure. The projectile creates a larger hole than its diameter at the front face of the PI-6 block. The velocity of the aerogels’ pressure wave was estimated to be approximately 930 m s^−1^ calculated using the aerogel’s Young’s modulus and bulk density. Therefore, the pressure wave velocity is higher than the projectile impact velocity (174 m s^−1^). Figure 10C and D show that the hole on the front face increased in width up to the point that the projectile was stopped. The diameter of the projectile nest inside the material is very close to the projectile diameter. Also, a circumferential crack was formed due to a tensile failure likely from a tensile stress generated by the projectile right after penetration.

**Figure 11:**
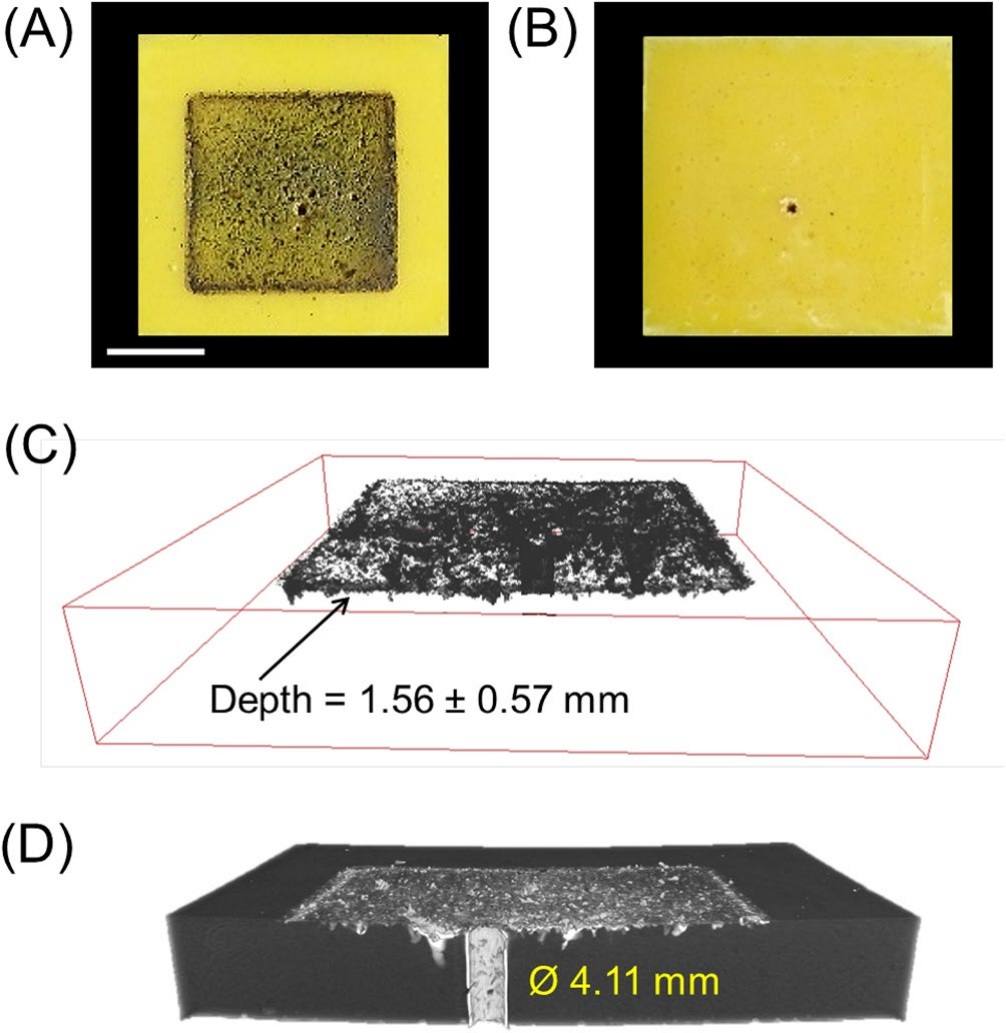
(**A**) The front and (**B**) the back faces of the PI-1 aerogel block after impact; (**C**) the µ-CT 3D rendering of the sabot pieces after penetrating on the front face of the PI-1 aerogel block; and (**D**) the µ-CT 3D rendering of the cross-section of the PI-1 impact zone. The scale bar is 1 in.

The aerogels’ material failure at high projectile impact velocities is different. As an example, the PI-1 aerogel block was impacted by a projectile with an impact velocity of 1283.5 m s^−1^. Figure 11A and B show the optical images taken from the front and back faces of the aerogel block, respectively. At this speed, the sabot also failed and was shattered into pieces by its impact on the sabot stopper, and therefore, the aerogel block experienced a secondary impact by the free-flying sabot pieces after the projectile impact. The black spots on the front face of the aerogel block are associated with the impacts from the sabot pieces (check Fig. 11A). The average penetration depth of the sabot-related impact was measured to be 1.56 mm from the µ-CT analysis of the impacted PI-1 aerogel block (see Fig. 11C). Movie S3 in the Supporting Information shows cross-sectional slices of the CT scanned volumetric image of the PI-1 sample. In contrast with the PI-6 sample, at high velocities, the aerogel’s material failure is a pure shear-off (an adiabatic shearing process). Figure 11D shows the µ-CT 3D rendering of the impacted aerogel block at the cross-section of the impact zone. It shows a neat cylindrical hole with a slightly bigger inner diameter than the projectile diameter.

**Figure 12:**
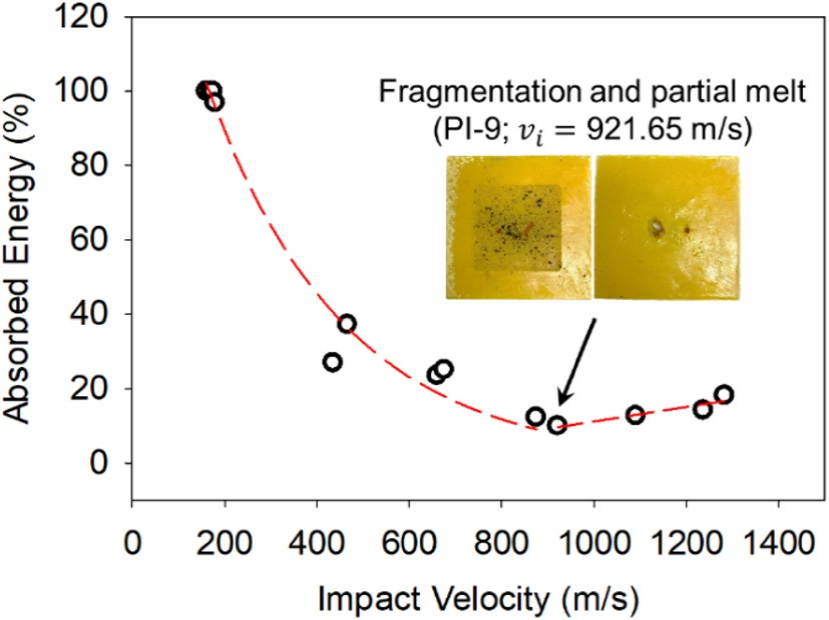
The ballistic energy absorption performance of the polyimide aerogel blocks as a function of the projectile impact velocity. The inset displays the front and back faces of the PI-9 aerogel block impacted by a projectile with a speed of 921.65 m s^−1^.

The overall ballistic energy absorption performance in terms of absorbed energy percentage by the impacted polyimide aerogel blocks as a function of the projectile impact velocity is shown in Fig. 12. Over the range of the projectile impact velocities in this study, the ballistic performance of the aerogel blocks exhibits two regimes. Initially, by increasing the projectile impact velocity up to about 900 m s^−1^, the absorbed energy drops as the projectile penetrates through the aerogel blocks without additional energy absorption by tensile stretching. After 900 m s^−1^, the absorbed energy increases slightly due to the aerogel blocks fragmentation and possibly a partial melting. The front and back faces of the PI-9 aerogel block impacted by a projectile with a speed of 921.65 m s^−1^ displaying the target fragmentation are shown in the inset of Fig. 12. Overall, the aerogels show a minimum 20% energy absorption capacity in this study.

## Experimental methods

### Materials

PMDA and DMBZ were purchased from Chriskev (Lenexa, KS). TEA and AA were purchased from Sigma Aldrich. BTC was purchased from Sigma-Aldrich. NMP, acetone, *tert*-butanol were purchased from Fisher Scientific. All materials were used as received. However, after short spans of atmospheric exposure, dianhydrides required vacuum-drying at 120 °C for 24 h.

### Synthesis

A polyimide aerogel was prepared using PMDA as the dianhydride, DMBZ as the diamine, and BTC as the crosslinker with a chain length of n = 40 and a polymer concentration of 10% w/w. The formulation was produced as follows. DMBZ (7.57 g, 35.67 mmol) was added to a solution of 115 ml NMP. This was stirred for approximately 15 min until the diamine was completely dissolved. PMDA (7.59 g, 34.80 mmol) was then added to this solution and stirred for 10 min until the dianhydride was completely dissolved. The solution turned a cloudy reddish-brown color when the dianhydride was added, and gradually turned to a transparent, pale yellow color. The acetic anhydride (26.32 ml, 278 mmol) and triethylamine (4.85 ml, 34.78 mmol) were added to the poly(amic) acid as catalysts to initiate imidization. This solution was stirred for approximately 10 min to completely incorporate the monomers for chemical imidizaiton, becoming extremely viscous after 5 min of stirring. The BTC (0.154 g, 0.58 mmol) cross-linker, previously dissolved in 7.57 ml NMP, was added to the polymer solution and stirred for 1–2 min at low rpm (150) to avoid creating air bubbles in the viscous solution, and then poured into molds to gel. Gelation occurred within 45 min. After gel synthesis and aging (24 h), the gel was solvent exchanged into *tert*-butanol by soaking it in five sequential baths of pure *tert*-butanol. The volume of the bath was approximately five times that of the gel, and solvent exchange was performed at approximately 40 °C. After the solvent exchange, the gel was transferred to an ambient-pressure drying chamber, where it was cooled below the freezing point of *tert*-butanol (i.e., 26 °C). At these conditions, the frozen *tert*-butanol within the gel sublimes rather than evaporating, thereby avoiding the damaging surface tension forces that arise at a liquid–vapor interface. A continuous flow of desiccated air was provided to the drying chamber, so that the *tert*-butanol concentration in the chamber was kept low, and the gel continued to dry via sublimation. The process continued until all the *tert*-butanol has been removed from the gel, at which point the dry aerogel was removed from the dryer and returned to ambient temperature.

### Material characterization

Attenuated total reflectance (ATR) infrared spectroscopy was conducted using a Nicolet Nexus 470 FT-IR spectrometer. Solid ^13^C NMR spectrum of the polyimide aerogel was obtained on a Bruker AVANCE-300 spectrometer with a 4 mm solid probe using cross-polarization and magic angle spinning at 11 kHz. Nitrogen sorption porosimetry was conducted on an ASAP 2000 surface area/pore size distribution analyzer (Micromeritics Instrument Corp.). Bulk densities were determined from the weight and the physical dimensions of the samples. Skeletal density was measured using an Accupyc 1340 helium pycnometer (Micromeritics Instrument Corp.). Thermal gravimetric analysis (TGA) was performed using a TA model 2950 HiRes instrument. Samples were run at a temperature ramp rate of 10 °C per min from room temperature to 750 °C under nitrogen or air. Scanning electron microscopy (SEM) was performed on a Hitachi S-4799-11 field emission scanning electron microscope using samples coated with gold/palladium. Quasi-static compression tests were performed on an Instron mechanical testing system (Instron Inc., Model 5969, Norwood, MA) with 500 N load cell (with the accuracy of 0.5% of the reading). The compression rate was set to 0.5 mm min^−1^. Cylindrical samples with 20 mm diameter and 12 mm height were used for the compression tests.

### Ballistic impact tests

The impact tests were carried out using a helium-filled gas gun connected to a vacuum chamber. The gun barrel is made of mild steel and has a 23 ft and 2-inch bore. A spherical steel projectile (hardened steel ball bearing) with 0.125 in diameter was placed in a cylindrical polycarbonate sabot. The helium in the pressure vessel was pressurized at the required pressure. The projectile and sabot were accelerated down the gun barrel by releasing the high-pressure helium using a burst disk consisting of a nichrome wire sandwiched between two or more Mylar® sheets, each at a nominal thickness of 0.005 in. The gun barrel protruded into the vacuum chamber which held the fixture for the specimens. Sabots were additively manufactured using a 3D printer (Markforged Onyx Pro, Markforged Inc., Watertown, MA). They were designed to increase specific stiffness. Sabots were printed with a carbon fiber infused nylon filament (Onyx, Markforged Inc., Watertown, MA) with bottom layers consisting of continuous fiberglass (Fiberglass, Markforged Inc., Watertown, MA) for added strength. The design decreased the overall mass of the accelerated package while being able to withstand the extreme acceleration. A conical orifice located at the end of the barrel captures and redirects the sabot material while allowing the projectile to continue along the flight path. The conical orifice also vents the remaining pressure in the gun radially outward to minimize forces on the target. The projectile impact velocity and exit velocity (in the case of penetration) were measured using two high-speed cameras (Photron SA-Z, Photron Inc., Tokyo, Japan). The cameras were calibrated before the impact test using an aluminum rod protruding from the gun barrel with calibration marks located at every inch. These cameras provided side views of the front and rear of the specimen. Also, a separate camera (Photron SA-Z, Photron Inc., Tokyo, Japan) was placed at the top of the front face of the specimen for a qualitative impact process investigation. The velocity of the projectile was measured by tracking the projectile’s center of mass position at five different locations before and after the impact. Calibration tests in which no panel was mounted indicated that the differences in velocity measurements between the two cameras were well under 1%. The cameras were operated in a continuous recording mode such that new images overwrote old images. The cameras were triggered in post-triggering mode: after the impact event was over, the camera was triggered to allow it to save the images acquired in few seconds prior triggering.

### X-ray micro-computed tomography

A Nikon X-ray CT system equipped with a 225 kV microfocus flat panel detector (Varex 2520DX) with a pixel matrix of 2000 × 2000 was used to observe the interior of the polyimide aerogels. The CT scan was performed on the impacted aerogel blocks. The X-ray energy and current parameters were set at 110 kV and 75 μA, respectively. The CT scans had an effective voxel size of 70.3 μm/voxel after reconstruction of 1080 projection images into the volumetric image. Finally, the volumetric image was loaded into ORS Dragonfly software for visualizations and post-processing.

## Conclusion

There is a need for advanced materials designed for orbital debris containment that are lightweight and low volume that offer particulate confinement, velocity deceleration, and energy dissipation. Current materials within a shield system for either containment during critical impact or energy absorption require complex structural supports and substantial mass and bulk density. This results in the need to reduce cost, weight, and volume together to combat the multiple issues with debris remediation. Herein, the ballistic performance of a class of lightweight and mechanically strong polyimide aerogels was studied through a series of impact tests at various velocities, from 150 to 1300 m s^−1^, using a helium-filled gas gun connected to a vacuum chamber and a spherical steel projectile. These aerogels showed a robust ballistic performance over the entire impact velocity range at a low areal density of just 0.2 g cm^−2^. For instance, the absorbed energy percentage was approximately 18% at the projectile’s impact velocity of 1283 m s^−1^. As a low-density impact-resistant material, these aerogels exhibit a great potential to be used as a stand-off spacing material in the stuffed Whipple shields to decelerate/capture secondary debris clouds. This would potentially lead to significant mass and volume savings in these shielding systems. Their other potential applications span areas with ballistic remediation requirements such as military helmets and shielding sensitive equipment.

## Supplementary Information

Supplementary Legends.

Supplementary Video 1.

Supplementary Video 2.

Supplementary Video 3.

## Data Availability

All data generated or analyzed during this study are included in this published article (and its Supplementary Information files).
