# Supplementary material for: Polyimide aerogels for ballistic impact protection
Source: Sci Rep. 2022 Aug 17;12:13933. doi: 10.1038/s41598-022-18247-z (PMC9385616; doi:10.1038/s41598-022-18247-z)
Supplement: Supplementary file 1 — Supplementary Legends. [file 41598_2022_18247_MOESM1_ESM.docx]

**Supporting Information**

The following files are available free of charge.

**Movie S1:** The footage associated with the impact event on the PI-6 aerogel (MP4).

**Movie S2:** Cross-sectional slices of the CT scanned volumetric image of the PI-6 sample (AVI).

**Movie S3:** Cross-sectional slices of the CT scanned volumetric image of the PI-1 sample (AVI).
